# *Paenibacillus polymyxa* 29-Y2: A Promising Endophytic Biocontrol Agent Against Wheat Common Bunt Caused by *Tilletia foetida*

**DOI:** 10.3390/plants15071072

**Published:** 2026-03-31

**Authors:** Zhiwei Wen, Niannian Yan, Xiaowei Guo, Qi Liu, Jing Chen

**Affiliations:** 1 Key Laboratory of the Pest Monitoring and Safety Control of Crops and Forests of the Xinjiang Uygur Autonomous Region, College of Agronomy, Xinjiang Agricultural University, Urumqi 830052, China; wen19945842141@163.com (Z.W.); 17690712604@163.com (N.Y.); guoxiaowei0208@163.com (X.G.); chenj@xjau.edu.cn (J.C.); 2 Key Laboratory of Prevention and Control of Invasive Alien Species in Agriculture & Forestry of the North-Western Desert Oasis, Ministry of Agriculture and Rural Affairs, Urumqi 830052, China

**Keywords:** wheat common bunt, biocontrol strain, *Paenibacillus polymyxa* 29-Y2, *Tilletia foetida Liro*, response surface methodology

## Abstract

Wheat common bunt, caused by *Tilletia foetida* Liro, is a devastating disease in wheat production. In this study, the antagonistic endophytic bacteria 29-Y2 were screened based on the germination rate of teliospore and the control effect of wheat common bunt. During primary screening, 29-Y2 had the best performance, with a 96.73% inhibition on TFL spore germination. In the deep screening, the control effect of 29-Y2 on wheat common bunt was 66.12% in pots. Based on morphological, physiological, and biochemical characteristics and molecular biological identification, the antagonist 29-Y2 was identified as *Paenibacillus polymyxa*. The antagonist 29-Y2 promoted the germination rate of wheat seeds and the growth of wheat seedlings at a solution dilution of 10^−5^ cfu/mL. In different field trials, the antagonists 29-Y2 both had better control efficiencies of 62.31% and 67.62% for wheat common bunt. In order to further promote the inhibition activities of 29-Y2, the optimal culture condition was 11.1 g/L of glucose, 20 g/L of yeast extract powder, 3.8 g/L of soybean pepyone and 10 g/L of NaCl based on the response surface methodology; the liquid loading volume was 15 mL, of which the inoculant amount accounted for 2%, the pH was 6.8, the temperature was 30 °C and the rotation speed was 186 r/min for 26 h. When the fermentation broth obtained under these cultivation conditions was diluted 10,000 times, the inhibition rate of TFL teliospore germination could reach 80.32%. The fermentation broth control effect in pots improved from 57.77% to 84.17%. It was a promising endophytic bacterium for the prevention and control of wheat common bunt.

## 1. Introduction

Wheat (*Triticum aestivum* L.) is a staple cereal crop of critical importance for global food security, particularly in China, where it serves as a major grain commodity [1]. Wheat common bunt, caused by *Tilletia foetida* Liro (TFL), is a globally prevalent devastating wheat disease. Infected wheat kernels develop distinctive grayish-brown pericarps encapsulating masses of dark teliospores, the primary survival and dispersal units of the pathogen [2,3]. These teliospores emit volatile trimethylamine compounds responsible for the characteristic fishy odor of diseased materials, rendering contaminated grains unmarketable and substantially reducing both nutritional quality and milling properties [4]. The disease is distributed across major wheat-producing regions worldwide and is also prevalent in China. It can cause yield losses ranging from 25% to 50%, and in severe cases up to 100%. Moreover, the toxic components also pose a threat to human and livestock health [5].

At present, the methods for controlling wheat common bunts are the use of pesticides, the breeding of resistant cultivars, timely planting and crop rotation [5,6]. In light of the issues of pesticide residue, strain resistance and environmental concerns associated with chemical control, biological control using beneficial microorganisms has emerged as a sustainable strategy [6]. Among these approaches, biocontrol bacteria are regarded as one of the most promising methods for pest control in the 21st century, owing to their superior colonization ability, environmental friendliness, and long-term protective effects. The biocontrol agents also exhibit multiple mechanisms of action, including growth promotion, pathogen antagonism, and the induction of plant resistance [7]. Through the optimization of the fermentation process via response surface methodology, the antifungal activity of the produced substances can be further enhanced, thereby establishing a basis for the development of highly effective microbial agents.

Although resistant varieties and pesticide application remain the main control strategies for wheat common bunt, there is an urgent need for environmentally friendly green control technologies, with biocontrol agents representing a highly promising approach for pathogen management. This study was aimed at screening and identifying endophytic *Paenibacillus polymyxa* strains that exhibited antagonistic activity against *Tilletia foetida*. The strains were characterized by using morphological, physiological–biochemical and molecular biological approaches. Additionally, the capacities of the spore germination inhibition rate, field control effect and growth-promoting effect were also systematically assessed. The results provide theoretical references for the green prevention and control of wheat diseases, and offer a guarantee for ensuring food security.

## 2. Results

### 2.1. Screening of Antagonistic Endophytes Against TFL

**Figure 1:**
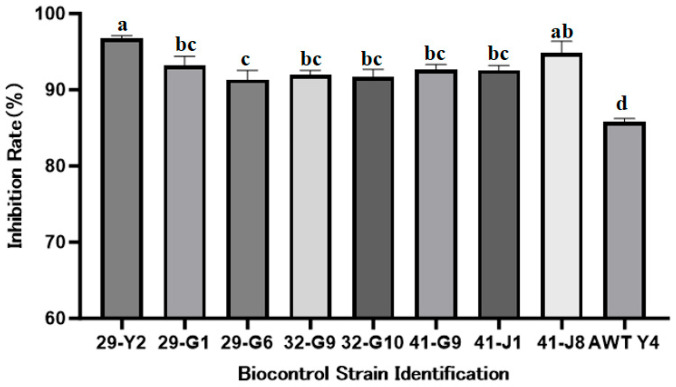
Initial screening revealed nine strains with inhibition rates exceeding 80%. Error bars indicate the standard deviation (SD) of the three replicates. Different lowercase letters indicate significant differences in the treatment, while the same letters indicate no significant differences (*p* < 0.05).

**Figure 2:**
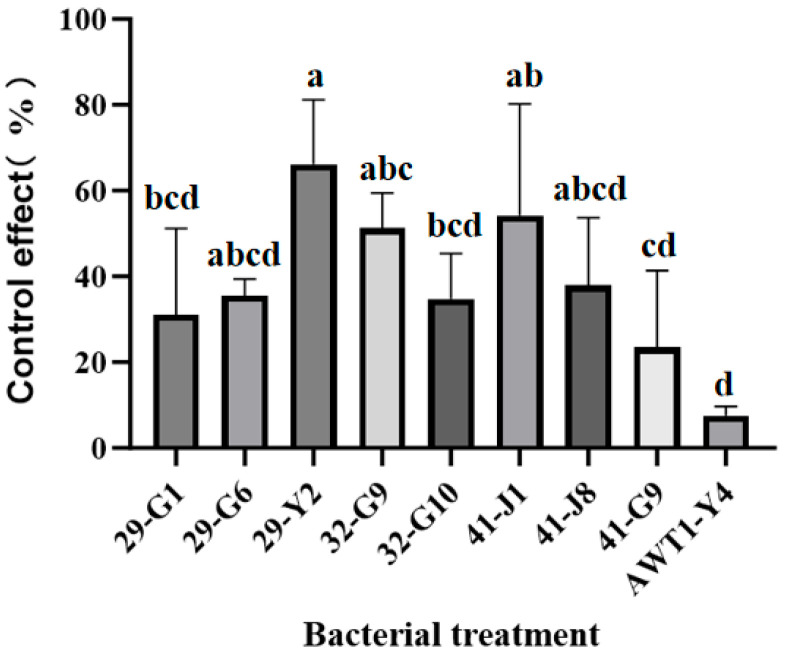
Deep screening of antagonistic strains for disease control in a greenhouse pot experiment. Error bars indicate the standard deviation (SD) of the three replicates. Different lowercase letters indicate significant differences in the treatment, while the same letters indicate no significant differences (*p* < 0.05).

In the initial screening, nine endophytic bacteria out of 120 strains were selected based on their teliospore germination inhibition rate exceeding 80% (Figure 1), among which strain 29-Y2 exhibited the highest inhibition rate of 96.73% ± 0.38. In the subsequent screening, these nine strains were evaluated in a greenhouse pot test for their efficacy against TFL. Strain 29-Y2 showed the highest control efficacy (66.12% ± 8.73), which was significantly (*p* < 0.05) higher than that of the other strains (Figure 2). Therefore, strain 29-Y2 was selected for further characterization.

### 2.2. Identification of Antagonistic Strain 29-Y2

#### 2.2.1. Morphological and Physiological Characteristics

**Figure 3:**
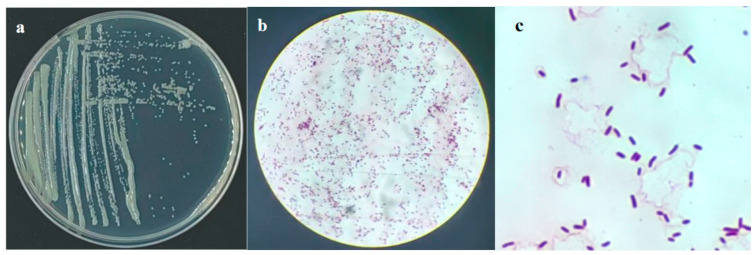
Morphological identification of strain 29-Y2. (**a**) Colony morphology; (**b**,**c**) Gram staining.

The colony of strain 29-Y2 on NA medium was circular, milky-white, convex, and opaque. The Gram reaction was positive (Figure 3).

The physiological and biochemical characterization of strain 29-Y2 is shown in Table 1. The strain exhibited motility in semi-solid agar. It tested positive for ornithine decarboxylase, lysine decarboxylase, Simmons’ citrate utilization, hydrogen sulfide production, and urease activity. In contrast, it yielded negative results for the Voges–Proskauer (VP) test, phenylalanine deamination, and acid production from mannitol, inositol, sorbitol, melibiose, ribitol, and raffinose.

#### 2.2.2. Molecular Identification

**Figure 4:**
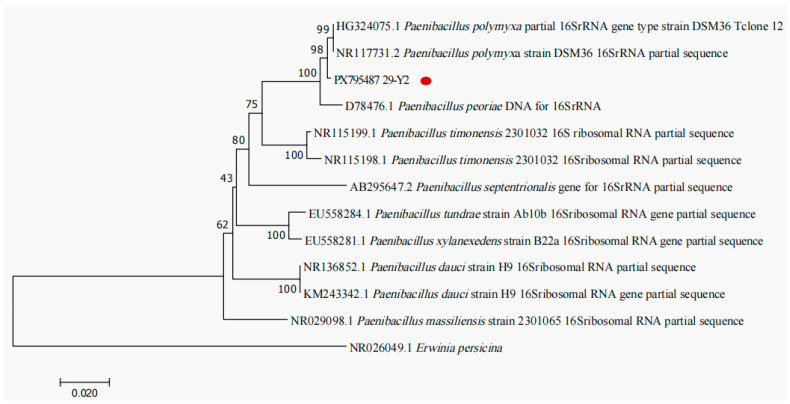
Phylogenetic tree of strain 29-Y2 based on 16S rDNA sequence. The red dot indicate the target strain.

As shown in Figure 4, the 16S rRNA gene sequence of the strain 29-Y2 (GenBank accession number PX795487) was arranged in a phylogenetic tree, which had the greatest similarity of 98% with *Paenibacillus polymyxa* (NR 117731.2, HG324075.1).

**Figure 5:**
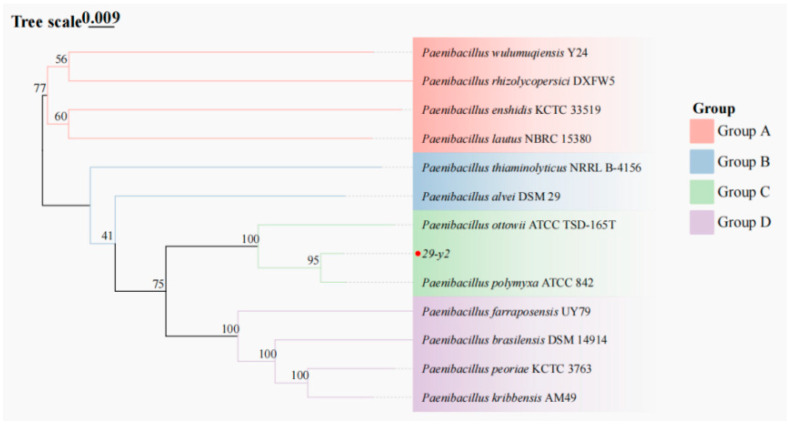
Phylogenetic tree of strain 29-Y2 based on whole-genome sequence. The red dot indicate the target strain.

The complete nucleotide sequence and a homology analysis of strain 29-Y2 were also performed, and the phylogenetic tree was constructed by using IQ-TREE2.4.0 software. As shown in Figure 5, strain 29-Y2 had the greatest similarity of 95% with *Paenibacillus polymyxa*.

Based on the morphological characteristics, physiological and biochemical analysis, and molecular identification, antagonistic strain 29-Y2 was identified as *Paenibacillus polymyxa*.

### 2.3. The Antifungal and Plant Growth-Promoting Potential of Strain 29-Y2

**Figure 6:**
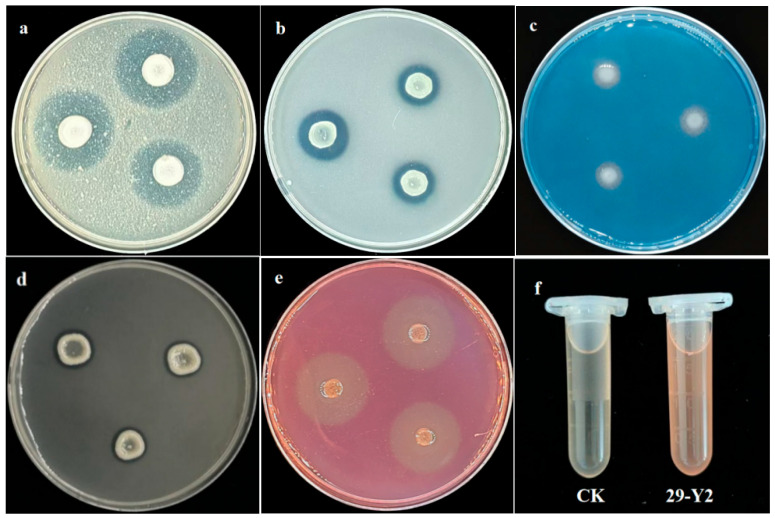
The plant growth-promoting potential of strain 29-Y2. (**a**) Protease; (**b**) organophosphorus; (**c**) siderophore; (**d**) amylase; (**e**) cellulase; (**f**) phytohormone.

The antifungal and plant growth-promoting potential performance of strain 29-Y2 was evaluated by characterizing several indicators on a plate. As shown in Figure 6, strain 29-Y2 had the potential to produce protease, organophosphorus, siderophore, amylase, cellulase and phytohormone.

### 2.4. Effect of Endophytic Bacteria 29-Y2 on Wheat Seed Germination

**Figure 7:**
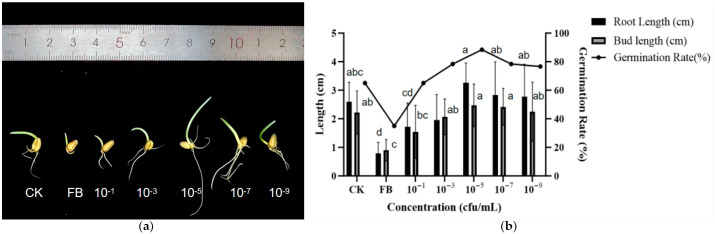
Effects of different dilution concentrations on wheat seed germination. (**a**) Comparison of different concentration treatments with sterile water; (**b**) the effect of gradient dilution of fermentation broth on wheat seeds (CK: control; FB: fermentation broth). Error bars indicate the standard deviation (SD) of the ten replicates. Different lowercase letters indicate significant differences in the treatment, while the same letters indicate no significant differences (*p* < 0.05).

The effect of endophytic bacteria 29-Y2 on wheat seed germination is presented in Figure 7. At a concentration of 10^−5^ cfu/mL, strain 29-Y2 had the greatest effect on the promotion of wheat seed germination, with a germination rate, root length, and bud length of 88.35% ± 7.26, 3.3 ± 0.8 cm, and 2.5 ± 0.7 cm, respectively.

### 2.5. Effect of Endophytic Bacteria 29-Y2 at Wheat Seedling Stage

**Figure 8:**
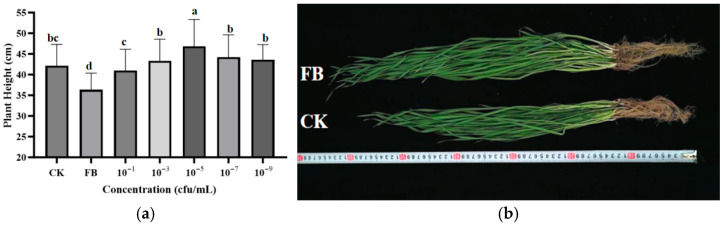
Effects of 29-Y2 on the growth of wheat at the seedling stage. (**a**) The effect of gradient dilution of fermentation broth on wheat; (**b**) comparison of fermentation broth treatment and sterile water treatment (CK: control group; FB: fermentation broth group). Error bars indicate the standard deviation (SD) of the three replicates. Different lowercase letters indicate significant differences in the presence of different concentration treatments (*p* < 0.05).

The effect of endophytic bacteria 29-Y2 on wheat seedling growth was presented in Figure 8. At a concentration of 10^−5^ cfu/mL, strain 29-Y2 had the greatest effect on the promotion of wheat seed germination, with a plant height of 46.8 ± 6.5 cm.

### 2.6. Field Efficacy of Strain 29-Y2 Against Wheat Common Bunt

**Figure 9:**
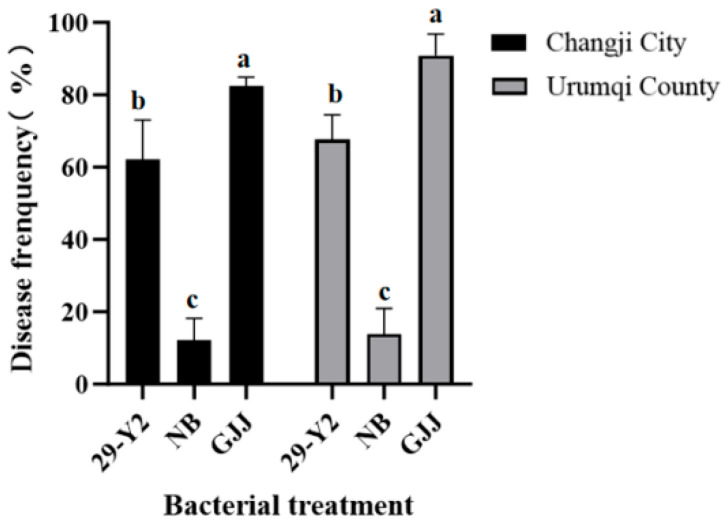
Field control efficacy at Changji City and Urumqi County. Error bars indicate the standard deviation (SD) of three replicates. Different lowercase letters indicate significant differences in the treatment, while the same letters indicate no significant differences (*p* < 0.05).

Field trials at two locations demonstrated that a root irrigation treatment eith the *Paenibacillus polymyxa* strain 29-Y2 fermentation broth significantly (*p* < 0.05) controlled wheat common bunt (Figure 9), with control efficacies of 62.31% ± 6.21 and 67.62% ± 3.96, respectively. Although the biocontrol treatment control effect was lower than the pesticide control treatment (fludioxonil), it still significantly improved the control effect compared with the control groups. So, strain 29-Y2 was a promising biocontrol agent and can be an alternative to chemical pesticides in the future.

### 2.7. The Optimal Culture Conditions for 29-Y2 Screened with Response Surface Methodology (RSM)

#### 2.7.1. Growth Curve Determination and Optimization Baseline Selection of 29-Y2

**Figure 10:**
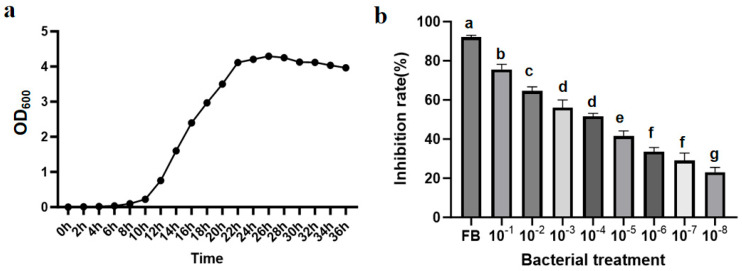
Growth curve and the optimization baseline selection of 29-Y2. (**a**) Growth curve; (**b**) inhibitory effects of different dilution concentrations on TFL. Error bars indicate the standard deviation (SD) of three replicates. Different lowercase letters indicate significant differences in the treatment, while the same letters indicate no significant differences (*p* < 0.05).

The growth curve of *Paenibacillus polymyxa* 29-Y2 and the dose–response relationship of its fermentation broth against TFL were determined. As shown in Figure 10a, biocontrol strain 29-Y2 entered the stationary phase at 22 h, reaching peak biomass (OD_600_ = 4.294) at 26 h. The teliospore inhibition rate assay revealed that the undiluted fermentation broth strongly inhibited the mycelial growth of TFL, with an inhibition rate of 91.14%. The half-maximal inhibitory concentration (IC_50_) was determined using the concentration at a 10^−4^ dilution of the fermentation broth. As shown in Figure 10b, with the fermentation broth dilution multiple increased, the antifungal activity decreased significantly.

#### 2.7.2. Screening of Medium

**Figure 11:**
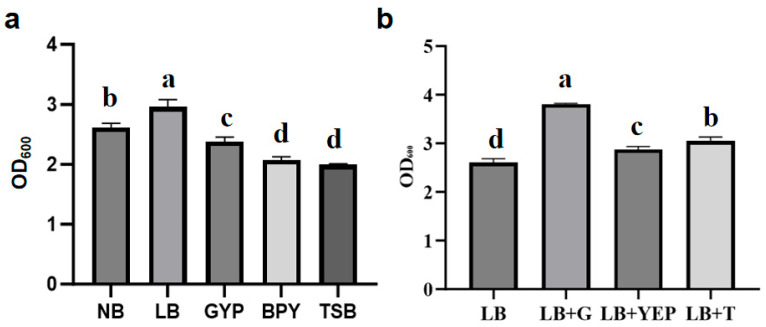
Optimization and screening results of culture medium and carbon source. (**a**) Screening of medium; (**b**) the influence of carbon sources on LB. (G: glucose; YEP: yeast extract powder; T: tryptone). Error bars indicate the standard deviation (SD) of three replicates. Different lowercase letters indicate significant differences in the treatment, while the same letters indicate no significant differences (*p* < 0.05).

As shown in Figure 11a, LB medium had the highest OD_600_ value of 2.958 out of five different media on the culturing of the biocontrol strain 29-Y2. Moreover, the glucose-supplemented medium as the carbon source had the highest OD_600_ of 3.808 (Figure 11b). As a result, LB with the glucose-supplemented medium was selected to be optimized in subsequent tests.

#### 2.7.3. Single-Factor Optimization

**Figure 12:**
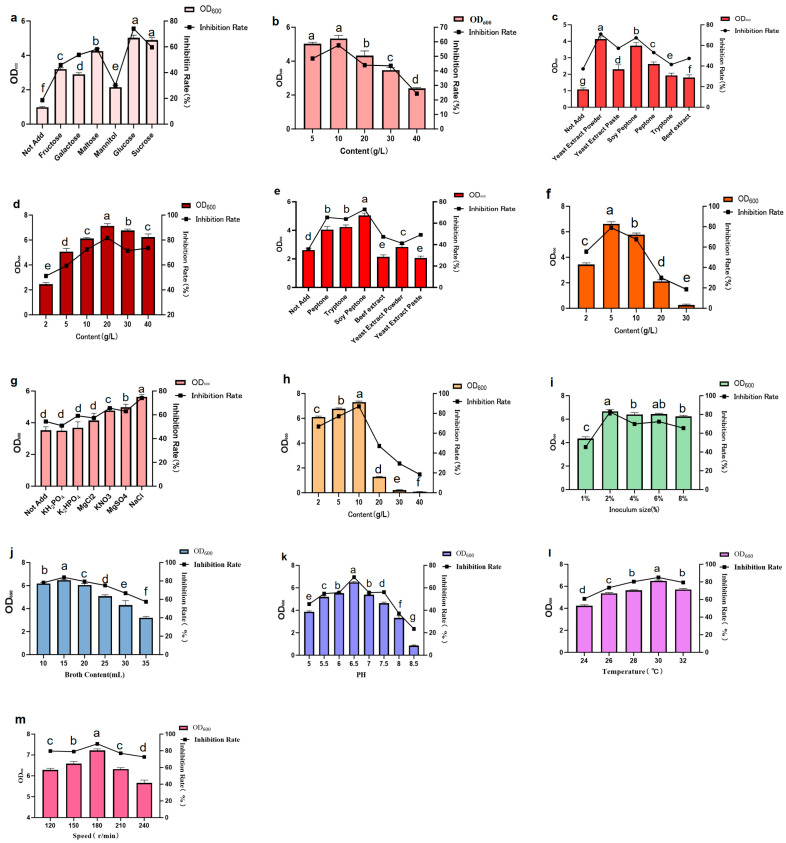
Single-factor screening results ((**a**) carbon source screening; (**b**) glucose content screening; (**c**) screening for replacement of yeast extract powder as nitrogen source; (**d**) yeast extract powder content screening; (**e**) screening for replacement of tryptone as nitrogen source; (**f**) soy peptone content screening; (**g**) inorganic salt screening; (**h**) NaCl content screening; (**i**) inoculum size screening; (**j**) broth content screening; (**k**) fermentation pH screening; (**l**) fermentation temperature screening; (**m**) fermentation rotation speed screening). Error bars indicate the standard deviation (SD) of three replicates. Different lowercase letters indicate significant differences in the treatment, while the same letters indicate no significant differences (*p* < 0.05).

Single-factor screening was performed by using the control variable method, and the optimal medium components were determined as follows: glucose (10 g/L) as the carbon source, yeast extract powder (20 g/L) and soy peptone (5 g/L) as the nitrogen sources, and sodium chloride (10 g/L) as the optimal inorganic salt. The optimal fermentation conditions were identified as follows: liquid loading volume of 15 mL, inoculum size of 2%, temperature of 30 °C, rotation speed of 180 r/min, and pH value of 6.5 (Figure 12).

#### 2.7.4. Screening of Significant Variables by the Plackett–Burman Design Experiment

Three positive factors (glucose, soy peptone, and rotation speed) were screened from nine single factors via the Plackett–Burman design and were selected for subsequent experiments (Table 2 and Table 3).

**Table 2:** Screening of significant variables using Placket–Burman design.

Code	A	B	C	D	E	F	G	H	I	OD_600_
1	−1	−1	1	−1	1	1	−1	1	1	9.35
2	1	1	1	−1	−1	−1	1	−1	1	10.45
3	1	−1	1	1	1	−1	−1	−1	1	8.14
4	1	−1	−1	−1	1	−1	1	1	−1	6.72
5	1	1	−1	−1	−1	1	−1	1	1	9.87
6	−1	−1	−1	1	1	−1	1	1	1	5.76
7	−1	1	−1	−1	−1	−1	−1	−1	−1	5.02
8	−1	1	1	−1	1	1	1	−1	−1	5.18
9	1	−1	1	1	−1	1	1	1	−1	6.55
10	−1	−1	−1	1	−1	1	1	−1	1	5.23
11	1	1	−1	1	1	1	−1	−1	−1	4.64
12	−1	1	1	1	−1	−1	−1	1	−1	5.92

**Table 3:** The results of the significance analysis for nine key factors.

Factors	Levels		F Test	*p* Value	Significance
	Low (−1)	High (1)			
Glucose/(g/L)	7	13	52.97	0.0184 *	3
Yeast extract powder/(g/L)	15	25	0.3539	0.6123	9
Soy peptone/(g/L)	3.5	6.5	37.60	0.0256 *	4
NaCl/(g/L)	7	13	57.78	0.0169 *	2
Broth content/(mL)	12.5	17.5	5.70	0.1397	6
Inoculum size/(%)	1.5	2.5	0.7638	0.4743	8
pH	6.3	6.7	5.02	0.1544	7
Temperature/°C	29	31	16.37	0.0560	5
Speed/(r/min)	160	200	117.66	0.0084 **	1

Note: * and ** indicate significant difference at 0.05 and 0.01 levels.

#### 2.7.5. The Box–Behnken Design with Significant Influencing Factors

The OD_600_ values derived from the Box–Behnken design are shown in Table 4; the numbers 1–17 represent the experimental groups. For the numbers in columns A, B, and C, −1, 0, and 1 represent the low, medium, and high levels, and the OD_600_ column represents the response value. The response surface could consist of 17 groups of OD_600_ values, which were analyzed in Design Expert software 8.0.6 using a regression equation fitted with multiple regression. The regression equation for the fermentation broth OD_600_ was derived as follows:

Y (OD_600_) = 9.32 + 1.15 × A + 0.5163 × B − 0.8613 × C − 0.7550 × A × B − 1.04 × A × C − 1.10 × B × C − 1.61 × A^2^ − 0.7588 × B^2^ − 2.04 × C^2^

The variance analysis results are shown in Table 5. The *p* value of this model was 0.0020, which was significantly due to it being less than 0.05. Meanwhile, the model types for A, C, A^2^, C^2^, AC, and BC were also significant.

**Table 5:** Variance analysis of regression equation.

Type	Sum of Squares	Degree of Freedom	Average of Squares	F Value	*p* Value	Significance
Model	63.87	9	7.10	11.59	0.0020	**
A	10.49	1	10.49	17.13	0.0044	**
B	2.13	1	2.13	3.48	0.1043	
C	5.93	1	5.93	9.69	0.0170	*
AB	2.28	1	2.28	3.72	0.0950	
AC	4.33	1	4.33	7.07	0.0326	*
BC	4.82	1	4.82	7.87	0.0263	*
A^2^	10.93	1	10.93	17.85	0.0039	**
B^2^	2.42	1	2.42	3.96	0.0869	
C^2^	17.50	1	17.50	28.58	0.0011	**
Residual	4.29	7	0.6123			
Lack-of-fit	2.78	3	0.9259	2.45	0.2029	
Pure error	1.51	4	0.3772			
Total error	68.15	16				

Note: F represents Fischer’s test; *p* represents probability; “*” indicates a significant effect (*p* < 0.05). “**” indicates a highly significant effect (*p* < 0.01).

#### 2.7.6. RSM Analysis and Determination of the Optimal Fermentation Conditions

**Figure 13:**
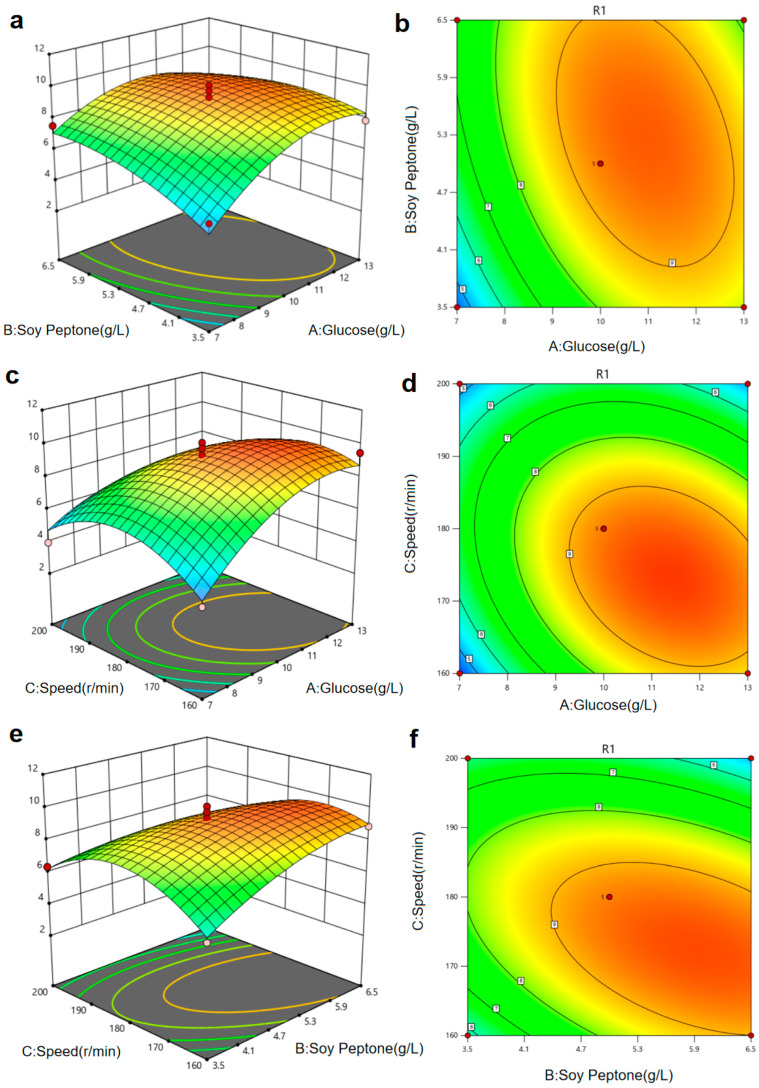
The RSM diagram and contour diagram results. (**a**,**b**) Glucose and soy peptone; (**c**,**d**) speed and glucose; (**e**,**f**) soy peptone and speed.

The RSM diagram and contour diagram between the factors when the number for OD_600_ was taken as the response value are shown in Figure 13a–f. It can be seen from Figure 13a,b that, when the glucose was constant, the number of viable bacteria first increased and then decreased with the extension of soy peptone. When the soy peptone was constant, with an increase in glucose, the number of viable bacteria also displayed a trend of first increasing and then decreasing. Figure 13c,d show that, when the speed was constant, the number of viable bacteria in 29-Y2 first increased and then decreased with the increase in Glucosea, and the vertex of the surface was the maximum point of the number of viable bacteria. Similarly, the surface of Figure 13e,f reflects the interaction between soy peptone, speed, and incubation time. In summary, there was a strong interaction between the factors.

The optimal fermentation medium for strain 29-Y2 was determined to be (in g/L) glucose 11.1, yeast extract 20, soy peptone 3.8, and NaCl 10. The corresponding culture conditions were established as follows: temperature at 30 °C, agitation speed of 186 rpm, initial pH of 6.5, a filling volume of 15 mL in the vessel, and an inoculum size of 2%.

**Figure 14:**
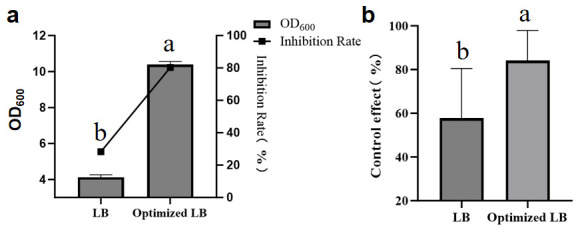
The comparison between LB and optimized LB. (**a**) OD_600_ and the inhibition rate. (**b**) The control effect in pot. Error bars indicate the standard deviation (SD) of three replicates. Different lowercase letters indicate significant differences in the treatment, while the same letters indicate no significant differences (*p* < 0.05).

As shown in Figure 14a,b, compared with the medium before, the optimized medium’s OD_600_ value improved from 4.12 to 10.38, the inhibition rate improved from 28.33% to 80.32%, and the control effect improved from 57.77% to 84.17%.

## 3. Discussion

The conventional management of wheat common bunt, caused by *Tilletia foetida* (TFL), has primarily relied upon two strategies: the application of chemical pesticides and the use of resistant wheat varieties. Chemical fungicides, including fludioxonil [8], fluoromethylbenzene [9] and carbendazim [10], remain highly effective, with average inhibition rates exceeding 80.0%, as previously reported. However, the sustained use of these fungicides has raised concerns regarding their environmental impact and the potential for resistance development. Concurrently, the identification and cultivation of resistant varieties constitute a fundamental long-term strategy. For example, Wang et al. [11] successfully identified nine resistant varieties. Recent advances in molecular breeding have begun to elucidate the genetic basis of resistance to *Tilletia* species. In one such study, Jia et al. [12] employed whole-genome resequencing combined with transcriptome analysis to identify candidate resistance genes against *Tilletia controversa* in wheat, including those involved in tryptophan metabolism and benzoxazinoid biosynthesis, thereby offering valuable targets for marker-assisted selection. Nevertheless, this approach is hindered by considerable challenges, such as lengthy breeding cycles, the risk of resistance breakdown due to pathogen evolution, and the difficulty of coupling robust resistance with desirable agronomic traits. In view of the limitations inherent to both chemical and genetic control strategies, the development and integration of sustainable biological alternatives in wheat common bunt management have become increasingly necessary.

Several biocontrol agents, such as *Pseudomonas* sp. MA 342 [13], have demonstrated a high efficacy against wheat common bunt diseases under field conditions. In this context, the study identified *Paenibacillus polymyxa* 29-Y2 as a potential endophytic antagonist, achieving a 96.73% inhibition of TFL teliospore germination in vitro, a 66.12% control efficacy in pot, and a 64.97% control in field. Beyond disease suppression, strain 29-Y2 also demonstrated significant plant growth-promoting effects, enhancing seed germination and seedling biomass at optimal concentrations (10^−5^ CFU/mL). Li et al. [14] demonstrated that *Paenibacillus polymyxa* WLY78 fixed atmospheric nitrogen via its *nif* gene cluster, providing up to 25.9% of the nitrogen nutrition for cucumber plants and significantly promoting their growth under low-nitrogen conditions. Furthermore, Li et al. [15] reported that *Paenibacillus polymyxa* ZYPP18 suppressed wheat sheath blight through the production of antimicrobial compounds (e.g., *polymyxin*, *iturin*, and *fusaricidin*) and promoted wheat growth via IAA synthesis and phosphate solubilization, enhancing root development and reducing disease incidence by up to 37.9% in field trials. In this study, the evaluation results showed that *Paenibacillus polymyxa* 29-Y2 was a highly promising candidate for the integrated biological management of wheat common bunt.

The fermentation conditions were profoundly influenced by the efficacy of the biocontrol agent. The optimization of medium composition and culture parameters was a common strategy for enhancing metabolite production and, consequently, disease suppression. The systematic optimization of fermentation to include medium composition and culture parameters significantly enhanced bacterial biomass and antibacterial activity for *Bacillus velezensis* F34-F44 [16], *Trichoderma harzianum* T-aloe [17] and *Paenibacillus polymyxa* CF05 [18]. Ran et al. [19] demonstrated that optimizing culture conditions significantly enhanced lipopeptide antibiotic production in *Paenibacillus polymyxa* 7F1, leading to potent antagonism against *Fusarium graminearum*, while transcriptomic analyses of *Tilletia controversa* interactions by Su et al. [20] revealed that pathogen infection perturbs host metabolic pathways, collectively reinforcing the rationale for optimizing fermentation conditions to produce metabolites that counteract pathogen-induced disruptions. Similarly, in this study, the optimization of the fermentation protocol for strain 29-Y2 resulted in an improved control efficiency against TFL through optimized medium and culture conditions.

Although this study optimized the fermentation conditions of 29-Y2 through RSM to obtain the culture formula and significantly improved the inhibition rate, the field control effect of the optimized formula still requires further verification. In addition, some research has shown that *Paenibacillus polymyxa* had a good preventive effect on *Puccinia striiformis* f. sp. *tritici* [21], *Fusarium oxysporum* f. sp. *lycopersici* [22] and *Fusarium moniliforme* [23], etc. *Paenibacillus polymyxa* 29-Y2 may also possess broad-spectrum antagonistic activity; however, the underlying mechanisms of disease resistance warrant further investigation. In conclusion, *Paenibacillus polymyxa* 29-Y2 emerged as a promising biocontrol candidate, providing a good biocontrol strain for controlling wheat common bunt.

## 4. Materials and Methods

### 4.1. Materials

Wheat kernels infected by TFL were collected from Huocheng County, Yining City, Xinjiang Uygur Autonomous Region. The TFL-susceptible wheat variety “Morocco” and 120 endophytic bacteria strains were preserved in the Plant Disease Epidemiology Laboratory, the College of Agronomy, Xinjiang Agricultural University.

### 4.2. Screening of Antagonistic Endophytic Bacteria

The endophytic bacterial strain was initially cultured on NA agar at 28 °C for 24 h and subsequently grown in NB liquid medium under shaken conditions (180 rpm, 28 °C) for an additional 24 h to produce the fermentation broth [21].

The initial screening was performed using the teliospore germination inhibition rate on water agar (2% *w*/*v*). The teliospore suspension concentration was set at 1 × 10^6^ spores/mL. Then, the mixture consisted of 100 µL bacterial fermentation broth and 100 µL teliospore suspension, which was cultured at 16 °C in darkness for 6 days; the control group was set as NB medium only with TFL. Teliospore germination was observed using an optical microscope. When the germ tube length of TFL exceeded the spore radius, the teliospores were determined to be germinated [10]. The strains which had an inhibition rate greater than 80% were selected for pot experiment screening. The inhibition rate was calculated as follows:

$$\mathrm{Inhibition}\;\mathrm{rate}\;=\;\frac{(C-T)}{C}\;\times \;100\%$$

C stands for the number of teliospore germinations in the control group; T stands for the number of teliospore germinations in the treatment group.

Prior to sowing, wheat seeds were coated with teliospores at a 20:1 (*w*/*w*) seed-to-inoculum ratio. Following seedling emergence, plants were treated via root drench with the fermentation broth of the selected antagonistic strains, while the control group received sterile water. All treatments, maintained in a growth chamber at 16 °C under a 16/8 h light/dark cycle with three replicates each, were assessed for disease incidence and control efficacy at the end of the growth period.

### 4.3. Identification of Antagonistic Strains

#### 4.3.1. Morphological and Physiological Characteristics

After incubating strain 29-Y2 on NA medium at 28 °C for 24–48 h, key colony features—such as color, size, shape, transparency, and edge uniformity, along with its Gram staining property—were examined under a microscope. Meanwhile, standard physiological and biochemical tests were performed as per the referenced manual [24].

#### 4.3.2. Molecular Biological Characteristics

The strain’s genomic DNA was isolated using a dedicated bacterial DNA extraction kit (TIANGEN, Beijing, China, DP302). For 16S rRNA gene amplification, PCR was run with universal primers 27F (5′-AGAGTTTGATCCTGGCTCAG-3′) and 1492R (5′-TACGGCTACCTTGTTACGACTT-3′) [25] under the specified conditions: initial denaturation at 95 °C for 4 min; 32 cycles of denaturation (94 °C, 20 s), annealing (55 °C, 20 s), and extension (72 °C, 90 s); and a final extension at 72 °C for 10 min. Successful amplification was confirmed through 1.0% agarose gel electrophoresis before the product was sequenced commercially by Sangon Biotech (Shanghai, China). The resulting sequence was compared to the GenBank database via BLAST (National Center for Biotechnology Information, National Library of Medicine, National Institutes of Health, Bethesda, MD, USA), and a phylogenetic tree was constructed employing the Neighbor-Joining method in MEGA 7.0.

In order to further confirm the taxonomic status of the strain and obtain its comprehensive genetic information, whole-genome sequencing was performed on the strain.

### 4.4. Analysis of Plant Growth-Promoting (PGP) Traits

The strain was screened for extracellular enzyme production (protease, cellulase, amylase) and other functional traits using established plate assays. Following Makhdoumi Kakhki et al. [26], hydrolytic activity was indicated by a color change on skim milk, carboxymethyl cellulose, or starch agar plates after 3–5 days of incubation at 28 °C. Siderophore production was analyzed on Chrome Azurol S (CAS) agar [27], with a color shift from blue to orange observed after 2–3 days at 28 °C. Indole-3-acetic acid (IAA) production was assessed on tryptophan-amended agar using Salkowski’s reagent overlay, where the development of a pink color indicated a positive result.

The abilities for phosphate solubilization, potassium solubilization, and nitrogen fixation were evaluated by using inorganic/organic phosphorus medium, Alexandrov’s medium, and Ashby’s medium, respectively [28]. All plates were cultured at 28 °C for 7 days, and positive resuwere was determined by the formation of dissolution halos.

### 4.5. The Effect of 29-Y2 on Wheat

In the seed treatment bioassay, surface-sterilized wheat seeds [8] were subjected to a dip in serial dilutions (10^−1^ to 10^−9^ cfu/mL) of the fermentation broth, whereas control seeds were soaked in sterile water. Each treatment comprised three replicates. Seed germination rate, root length, and shoot length were evaluated following 48 h of incubation at 28 °C. Subsequently, a pot trial was performed to assess growth promotion: ten treated seeds per pot (three replicate pots) were cultivated under greenhouse conditions for one month, after which the plant height was determined.

### 4.6. The Field Control Effect of 29-Y2 on TFL

From 2024 to 2025, a trial application of the control effect of strain 29-Y2 on TFL was carried out in the experimental fields in Changji City and Urumqi County. The experimental design was a randomized complete block with three replications. The treatments were: (1) water control group (root drench), (2) nutrient broth control group (root drench), (3) pesticide control group (seed dressing with 24 mL/L fludioxonil), and (4) biocontrol control treatment (root irrigation with a 50× dilution of 29-Y2 fermentation broth). Diseased Survey was carried out in the wheat full ripening stage, and the field control effect was calculated as follows:

$$\mathrm{control}\;\mathrm{effect}(\%)\;=\;\frac{C\;-\;T}{C}\;\times \;100$$

C stands for the amount of diseased wheat in the control group; T stands for the amount of diseased wheat in the treatment group.

### 4.7. Optimizing the Culture Conditions for 29-Y2 with RSM

#### 4.7.1. Growth Curve of 29-Y2 and Inhibitory Effects of Different Dilution Concentrations on TFL

To determine the optimal cultivation time for strain 29-Y2, the growth curve was measured using spectrophotometry. The individual bacterial colony was transferred to NB culture medium to obtain the fermentation broth under the condition of shaking at 28 °C, 180 r/min for 24 h, and then cultured in LB medium. Growth curves were generated by measuring OD_600_ at 2 h intervals against a sterile uninoculated medium control, with each treatment conducted in triplicate. When the growth curves achieved the stationary phase, the fermentation broths of strain 29-Y2 were diluted at a gradient (10^−1^ to 10^−8^) to evaluate the inhibitory effects. The half-maximal inhibitory concentration (IC_50_) was determined by fitting a dose–response curve (inhibit a specific biological activity by 50%) to log-transformed concentration data.

#### 4.7.2. The Screening of Optimal Fermentation Medium

To screen the optimal medium for subsequent optimization, strain 29-Y2 was cultured in five different media (NB, LB, BPY, GYP, TSB) under standardized conditions (28 °C, 180 rpm, 24 h) with three replicates and evaluated based on OD_600_ measurements.

Furthermore, the screened LB medium formulation lacked a primary carbon source. Thus, equal amounts of glucose, yeast extract powder, and tryptone were supplemented to the original LB formula to determine the influence of carbon sources.

#### 4.7.3. Single-Factor Method for Determining the Optimal Components

Single-factor experiments were conducted to screen and optimize the most suitable carbon source, nitrogen source, and inorganic salt and their optimal addition amounts for the strain’s fermentation medium. The fixed fermentation conditions were a 1% (*w*/*w*) inoculation of fermentation broths, 28 °C, and 180 r/min for 24 h, with a 20 mL broth content. The standard LB liquid medium was used as the control. Then, different carbon sources (glucose, sucrose, fructose, maltose, galactose, and mannitol) were added in equal amounts (10 g/L) to prepare liquid fermentation media with different carbon sources. After inoculation, the OD_600_ value of the liquid fermentation medium was measured; the growth rate of strain 29-Y2 and the inhibition rate on TFL teliospores were taken as comprehensive evaluation indicators for the optimal culture formula.

For carbon source screening, based on the LB medium, different carbon sourcewereas tested at varying concentrations (5, 10, 20, 30, 40 g/L). The most suitable carbon source and its optimal concentration were determined by measuring the teliospore germination inhibition rate. For nitrogen source screening, beef extract, peptone, and soy peptone were used in equal amounts to replace tryptone and yeast extract in the LB medium. The same concentration gradient (2, 5, 10, 20, 30, 40 g/L) and fermentation conditions as in carbon source screening were applied to identify the optimal nitrogen source and its concentration. For inorganic salt screening, MgCl_2_, K_2_HPO_4_, KH_2_PO_4_, MgSO_4_, and KNO_3_ were used in equal amounts (10.0 g/L each) to replace NaCl in the LB medium, following the same concentration gradient (2, 5, 10, 20, 30, 40 g/L) and fermentation conditions. The most suitable inorganic salt and its optimal concentration were selected. The optimal fermentation conditions were conducted as follows: broth content (10, 15, 20, 25, 30, 35 mL), inoculum size (1%, 2%, 4%, 6%, 8%), pH (5, 5.5, 6, 6.5, 7, 7.5, 8, 8.5), fermentation temperature (24, 26, 28, 30, 32 °C), and fermentation speed (120, 150, 180, 210, 240 r/min).

#### 4.7.4. Screening of Significant Variables with Placket–Burman Design

In this test, process variable optimization was performed by using the PB design in Design-Expert 7.0. Nine independent impact factors (glucose, yeast extract powder, soy peptone, NaCl, broth content, inoculum size, pH, temperature and speed) were investigated to identify the variables that significantly affect the antimicrobial activity (Table 6). Each variable was divided into two levels: “1” and “−1”. The OD_600_ (Y) was set as the response value. The tests were conducted with three replicates.

#### 4.7.5. Optimization of the Culture Conditions with Box–Behnken Design

Utilizing the three key factors (glucose, soy peptone, and speed) identified from the PB design, a Box–Behnken design with three levels (high (+1), medium (0), and low (−1)) was applied, and the response surface methodology was employed via Design-Expert 7.0 to model and determine the optimal cultivation conditions with triplicate validation (Table 7).

### 4.8. Statistical Analysis

All statistical analyses were performed using IBM SPSS Statistics (version 22.0; IBM Corp., Armonk, NY, USA). Statistical analysis was performed on data obtained from independent experiments. A one-way analysis of variance (ANOVA) was conducted, and, where significant differences were found, group means were compared using the Waller–Duncan K-ratio *t*-test at a 0.05 significance level. Results are presented as mean ± standard deviation (SD). Data visualization, including the preparation of graphs and charts, was conducted using GraphPad Prism (version 10.1.2; GraphPad Software, San Diego, CA, USA) and Microsoft Excel 2019 (Microsoft Corp., Redmond, WA, USA). Sequence alignments were performed using MEGA 7.0 and IQ-TREE, which constructed phylogenetic trees.

## 5. Conclusions

This study successfully isolated and identified the endophytic bacterium *Paenibacillus polymyxa* 29-Y2 as a potent dual-functional agent. The strain exhibited strong in vitro antagonism against *Tilletia foetida* teliospore germination and provided effective control of wheat common bunt in both greenhouse and field trials, alongside significant plant growth-promoting effects. Through response surface methodology optimization, the ideal fermentation medium was derived. This optimized process markedly enhanced the bacterial yield and antifungal activity, and the fermentation broth control effect was improved. These results collectively underscore the significant potential of *Paenibacillus polymyxa* 29-Y2 as a promising biocontrol agent and biofertilizer for the integrated management of wheat common bunt.

## Figures and Tables

**Table 1:** Physiological and biochemical characteristics of 29-y2.

Physiological and Biochemical Traits	Results	Physiological and Biochemical Traits	Results
Semi-solid agar	+	MR-VP	−
Ornithine decarboxylase broth	+	Phenylalanine	−
Lysine decarboxylase broth	+	Mannitol	−
Amino acid decarboxylase broth	−	Inositol	−
Simmons’ citrate	+	Sorbitol	−
Hydrogen sulfide	+	Melibiose	−
Urease	+	Ribitol	−
Peptone water	−	Raffinose	−

Note: “+” represents positive and “−” represents negative.

**Table 4:** The Box–Behnken design results.

Numbers	A	B	C	OD_600_
1	−1	1	0	7.54
2	−1	−1	0	5.16
3	1	0	1	5.25
4	1	−1	0	7.87
5	0	1	1	5.37
6	0	−1	1	6.37
7	0	0	0	8.89
8	−1	0	1	3.95
9	0	0	0	8.56
10	−1	0	−1	4.01
11	0	0	0	10.08
12	1	1	0	7.23
13	0	0	0	9.34
14	0	0	0	9.73
15	0	−1	−1	5.48
16	1	0	−1	9.47
17	0	1	−1	8.87

**Table 6:** Fermentation factor and level of 29-Y2.

Variables	Code	Level		
		−1	0	1
Glucose/(g/L)	A	7	10	13
Yeast extract powder/(g/L)	B	15	20	25
Soy peptone/(g/L)	C	3.5	5	6.5
NaCl/(g/L)	D	7	10	13
Broth content/(mL)	E	12.5	15	17.5
Inoculum size/(%)	F	1.5	2	2.5
pH	G	6.3	6.5	6.7
Temperature/°C	H	29	30	31
Speed/(r/min)	I	160	180	200

**Table 7:** Variables and levels for Box–Behnken design of 29-Y2.

Variables	Code	−1	0	1
Glucose/(g/L)	A	7	10	13
Soy peptone/(g/L)	B	3.5	5	6.5
Speed/(r/min)	C	160	180	200

## Data Availability

The original contributions presented in this study are included in the article. Further inquiries can be directed to the corresponding author.
